# Temozolomide Induced Hypermutation in Glioma: Evolutionary Mechanisms and Therapeutic Opportunities

**DOI:** 10.3389/fonc.2019.00041

**Published:** 2019-02-04

**Authors:** Paul Daniel, Siham Sabri, Ahmad Chaddad, Brian Meehan, Bertrand Jean-Claude, Janusz Rak, Bassam S. Abdulkarim

**Affiliations:** ^1^Division of Radiation Oncology, Department of Oncology, McGill University, Montréal, QC, Canada; ^2^Department of Pathology, McGill University, Montréal, QC, Canada; ^3^Research Institute of the McGill University Health Centre, Montréal, QC, Canada; ^4^The Laboratory for Imagery, Vision and Artificial Intelligence, École de Technologie Supérieure, Montréal, QC, Canada; ^5^Department of Pediatrics, McGill University, Montréal, QC, Canada; ^6^Department of Medicine, McGill University, Montréal, QC, Canada

**Keywords:** glioma, recurrence, MGMT, hypermutation, temozolomide

## Abstract

Glioma are the most common type of malignant brain tumor, with glioblastoma (GBM) representing the most common and most lethal type of glioma. Surgical resection followed by radiotherapy and chemotherapy using the alkylating agent Temozolomide (TMZ) remain the mainstay of treatment for glioma. While this multimodal regimen is sufficient to temporarily eliminate the bulk of the tumor mass, recurrence is inevitable and often poses major challenges for clinical management due to treatment resistance and failure to respond to targeted therapies. Improved tumor profiling capacity has enabled characterization of the genomic landscape of gliomas with the overarching goal to identify clinically relevant subtypes and inform treatment decisions. Increased tumor mutational load has been shown to correlate with higher levels of neoantigens and is indicative of the potential to induce a durable response to immunotherapy. Following treatment with TMZ, a subset of glioma has been identified to recur with increased tumor mutational load. These hypermutant recurrent glioma represent a subtype of recurrence with unique molecular vulnerabilities. In this review, we will elaborate on the current knowledge regarding the evolution of hypermutation in gliomas and the potential therapeutic opportunities that arise with TMZ-induced hypermutation in gliomas.

## Introduction

Glioma refers to a group of malignant brain tumors comprised of oligodendroglioma, anaplastic astrocytoma, and glioblastoma (GBM) (1). Amongst gliomas, GBM is the most commonly diagnosed malignant primary brain tumor making up 54% of all gliomas and 16% of all primary brain tumors (2). GBM is also the most lethal brain tumor with a median survival of only 15 months (2–4). GBM can be further stratified into IDH1 wild-type (90%) and IDH1 mutant (10%). Patients with IDH1 mutations are thought to comprise secondary GBM as they are enriched for ATRX mutations similar to that found in lower-grade glioma and patients survive for a median survival of 31 months which is consistent with lower-grade glioma (5).

The emergence of next-generation sequencing and characterization of the genome, epigenome, and transcriptome of GBMs revealed an increasing level of disease complexity. For example, it is now known that GBM is not a homogenous disease but comprises 3 distinct subtypes; Proneural (PN), Mesenchymal (MES), and Classical (CL) which are able to classify both primary and recurrent disease (6–9). Comparison of these molecular subtypes shows that each subtype is enriched for unique molecular alterations. PN is enriched for aberrations in the gene expression of platelet-derived growth factor receptor alpha (PDGFRA) and *TP53* mutations, whilst MES and CL are enriched for neurofibromatosis type I (NF1), and epidermal growth factor receptor (EGFR) alterations, respectively (8, 9). Similarly, each subtype has also been found to be associated with a specific local immune tumor microenvironment. By comparing immune signatures between each subtype, MES tumors were found to be enriched for macrophages and neutrophil signatures (6). In contrast, PN tumors exhibit suppression of CD4^+^ T-cell signature while CL tumors were enriched for dendritic cell signatures (6). Studies showing that an individual tumor is comprised of cells from multiple subtypes (i.e., intra-tumoral heterogeneity) have added additional complexity to this picture of heterogeneity (10, 11).

The current treatment regimen for primary GBM implemented since 2005 involves surgical resection followed by concurrent chemoradiation (12). This aggressive upfront trimodal regimen improved 2-years survival from 10% for treatment with radiotherapy (RT) alone to 27% compared to treatment with RT and Temozolomide (TMZ) (12). Despite this improvement, recurrence following treatment remains inevitable, typically occurring within months following completion of treatment at first diagnosis and is ultimately lethal as recurrent disease shows limited response to further chemoradiation. To date, there are currently no known therapies, which provide substantial survival benefit to GBM patients at recurrence, urging investigation into alternative treatment options. Understanding the mechanisms underlying response and emergent resistance to chemotherapy is therefore of utmost importance to inform decisions about the next generation of therapies.

## The Cytotoxic Effect of Temozolomide

**Figure 1:**
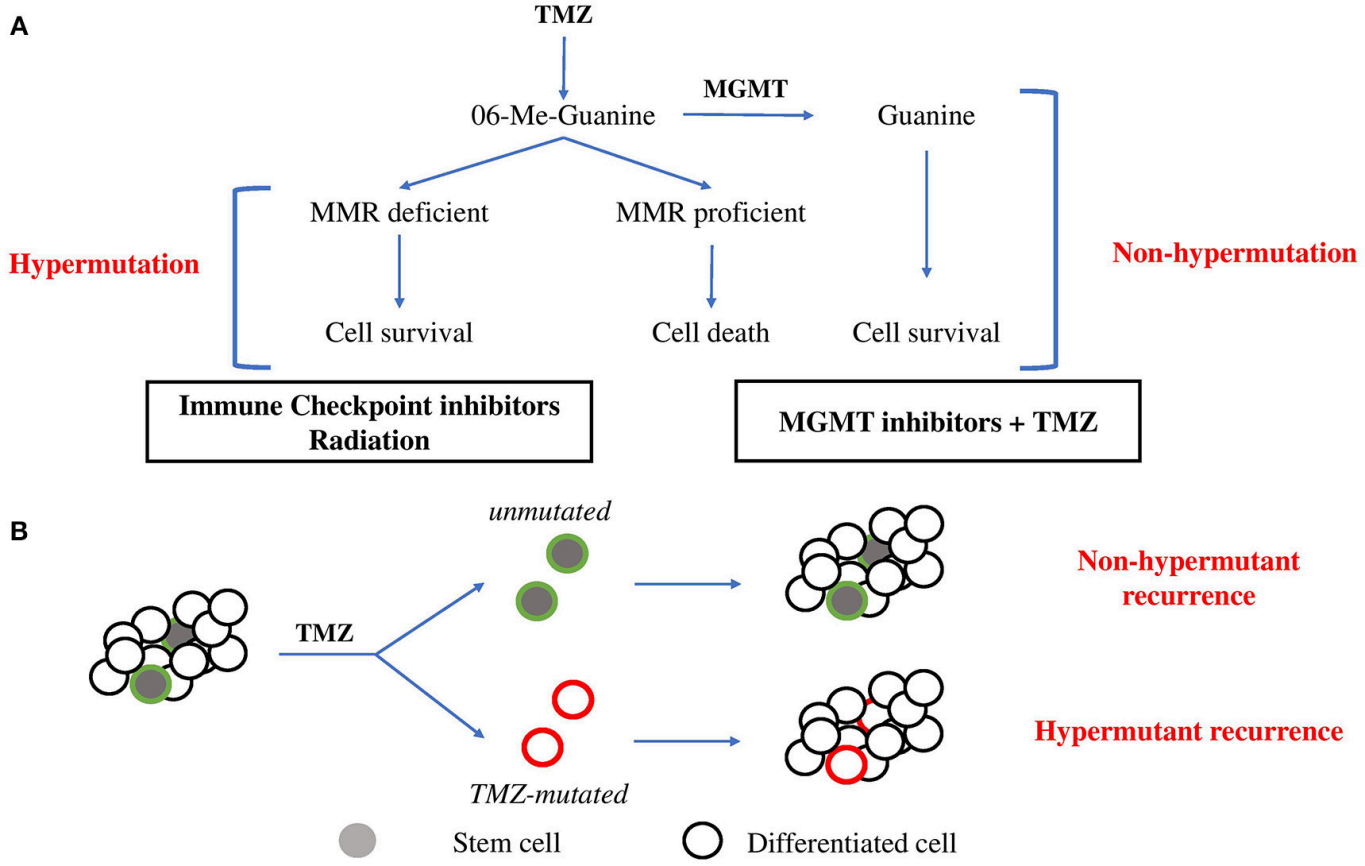
Evolutionary pathways toward hypermutation. **(A)** 06-Me-Guanine are generated through TMZ exposure. In the presence of MGMT, methyl adducts are removed and the cell survives without gain in mutagenesis. In the absence of MGMT, MMR status determines survival. In MMR proficient cells, futile repair leads to double strand breaks and cell death. In cells which lose MMR proficiency, cells gain tolerance to base mismatch and cells acquire genomic hypermutation. Suggested therapies are listed below. **(B)** Stem cell hierarchy of tumor growth may provide an alternate means of resistance to hypermutation. Minor populations of stem cells maintain tumor growth through differentiation. Upon exposure to TMZ, stem cells may be minimally affected by chemotherapy due to greater drug efflux activity and slower proliferation rate and so repopulate tumor mass with non-hypermutant progeny. Alternatively, stem cells which acquire hypermutation will give rise to hypermutant recurrent tumors.

TMZ, the main chemotherapy utilized for glioma, is an alkylating pro-drug which methylates DNA at the O^6^ position of guanine (13). During DNA replication, the maintenance of this methyl-adduct causes a mismatch pairing of guanine with thymine rather than cytosine leading to genomic instability and eventually cell death (13, 14). Two major mechanisms oppose the cytotoxic action of TMZ (Figure 1A). O^6^ methylguanine-DNA methyltransferase (MGMT), a suicide enzyme able to sequester the methyl-adduct from O^6^ guanine through covalent transfer, effectively repairs the alteration prior to replication (15). Consistent with this role for MGMT in driving resistance, *MGMT* promoter methylation, which is an indirect measure of the ability for cells to express the MGMT protein, is one of the strongest predictors of response to TMZ. Comparison of patient cohorts treated with chemoradiation revealed that those with MGMT methylation survive a median on 21.7 months compared to a median survival of just 15.3 months for patients without MGMT methylation (16).

In the absence of MGMT expression, resultant base mismatches invoke the mismatch repair (MMR) pathway. MMR proteins including MSH2, MSH6, MLH1, and PMS2 recognize and bind to the mismatched guanine and cause cells to enter a cycle of DNA repair (13, 17). Mismatches in newly synthesized daughter DNA strands are repaired whilst methyl adducts persist on parental DNA in the absence of MGMT. This leads to a cycle of futile repair followed by mismatching which eventually induces DNA double strand break formation, cell arrest and death (13). MMR capacity is therefore essential to repairing TMZ-induced toxicity. Consistent with this, comparison of MMR protein expression in 80 matched primary and recurrent GBM specimens treated with chemoradiation revealed consistent downregulation of MMR repair genes in recurrent GBM, highlighting the importance of MMR in dictating response to TMZ (18).

## Recurrent Glioma and Emergence of Hypermutation

The failure of chemoradiation, culminating with the inevitability of recurrence and acquisition of a chemo-resistant phenotype have spurred investigations into novel approaches toward treating recurrent disease. An emerging paradigm for finding effective treatments for recurrent GBM is targeted therapy, where treatment is specifically directed against driver alterations necessary for maintenance of malignant phenotypes. Increased availability and reduced cost of sequencing allowed interrogation of the molecular landscape of disease and identification of clinically relevant “subtypes” spurring further interest in targeted therapies. Notably, subtyping of disease states has been a major advancement in simplifying inter-tumoral heterogeneity whilst facilitating the identification of a targetable subset of patients sharing common molecular features. A recent remarkable finding from several longitudinal observational studies comparing pre- and post-treatment glioma has now established that at least two distinct genomic outcomes exist at recurrence; *hypermutant* and *non-hypermutant* recurrence (7, 19–23). For hypermutant recurrent tumors, the hallmark identifiers include (i) fold increases in subclonal mutations across the whole genome, (ii) enrichment of the C:G>T:A mutational signature indicative of TMZ mutagenesis, and (iii) gain of inactivating mutations in MMR pathway components (7). In comparison, non-hypermutant recurrent tumors do not exhibit any of these features but instead maintain a similar level of tumor mutational burden (TMB) compared to the primary tumor. Greater understanding of the processes which dictate emergence of these subtypes and the underlying molecular mechanisms responsible for maintenance of malignant features will likely be essential for the identification of targeted therapies against each subtype.

Attempts to understand the processes responsible for emergence of a hypermutant state have thus far been limited to observational studies of glioma, from primary to recurrent states. Although initially observed to be associated with malignant transformation of low-grade to high-grade glioma, TMZ-induced hypermutation has now been observed to occur in grade IV GBM, albeit occurring at a lower frequency compared to low grade (19, 21, 22). Specifically, recent reports from the largest observation study to date found that whilst only ~10% of GBM display hypermutation at recurrence, a much higher proportion of low grade gliomas emerge as hypermutant following treatment with TMZ (7, 22). This differential capacity for low and high-grade glioma to evolve toward hypermutation raises several questions related to the exact mechanisms which dictate predisposition to undergo mutagenesis.

## O-6-Methylguanine-DNA Methyltransferase (MGMT)

To date, *MGMT* promoter methylation is the strongest correlative feature which predicts GBM hypermutation at recurrence (7). Unsurprisingly, the importance of MGMT in preventing mismatch pairing during DNA replication due to removal of methyl adducts is likely responsible for this relationship to hypermutation. It is interesting to note however that reports vary regarding the correlation between MGMT protein expression and *MGMT* promoter methylation, making it plausible that methylation status of *MGMT* may play a surrogate role as a biomarker of a state predisposed toward hypermutation (24, 25). One potential alternative is the observation that *MGMT* promoter methylation may be indicative of a global hypermethylated phenotype. Indeed, epigenetic features have now been found to impact the ability for DNA repair to take place, as evidenced by the differential rates of mutagenesis predicated by chromatin accessibility (26). Supporting the impact that epigenetic features can have upon hypermutation, low grade gliomas exhibit a global higher hypermethylated state compared to GBM whilst undergoing hypermutation at a much higher frequency despite exhibiting similar proportions of *MGMT* promoter methylation at first diagnosis (27). Whether hypermethylated subtypes of GBM, defined by IDH1 mutation and a global CpG island methylation phenotype G-CIMP, undergo hypermutation at a higher rate than IDH1 wild-type non-G-CIMP GBM, which are comparatively hypomethylated is not yet known. This would provide critical information regarding the role that epigenetic status plays in dictating the emergence of hypermutation (28).

## Mismatch Repair Proteins

Along with MGMT methylation, acquired mutations in MMR genes have been strongly correlated with hypermutation (7, 19, 21). As described earlier, MMR represents the main mechanism by which mismatched bases are repaired and downregulation of these genes is a common mechanism by which gliomas acquire resistance to TMZ. Indeed, minimal loss in MMR genes MSH2 and MSH6 have been shown to be sufficient to provide substantial survival benefit to cells (29). It is important to note however that whilst MMR gene downregulation is common to recurrent glioma and may represent a convergent mechanism of acquired chemoresistance, MMR pathway *mutation* seems specifically enriched in hypermutant recurrence(7).

One explanation for why this may occur is the observation that MMR proteins are involved in alternate repair pathways which may contribute to resistance to mutagenesis. For example, non-homologous end joining (NHEJ) and homologous recombination (HR) are alternative DNA repair pathways which may drive repair of double strand breaks (DSB) following TMZ treatment. The MMR protein MSH6 directly binds to KU70, a regulatory subunit of the DNA-dependant protein kinase involved in NHEJ and HR repair (30). Similarly, MSH2 is a critical component of the BRCA1 associated genome surveillance complex that recognizes and initiates response to abnormal DNA structures (31). Consistent with the role of MMR is alternate DNA repair pathways, MSH6 knockout cells display impaired NHEJ typified by accumulation of persistent DSBs (30). Together these findings suggest that the complete loss of function of MMR proteins may be necessary for reduced capacity to repair DNA defects, allowing emergence of hypermutation.

## Additional Influences Upon the Emergence of Hypermutation

The majority of GBM tumors will recur as non-hypermutant displaying no overt signs of widespread mutagenesis following TMZ treatment (7). This occurs despite MGMT expression being reportedly stable between primary and recurrent disease (32), suggesting additional features are responsible for protecting from the mutagenesis imposed by TMZ. TMZ cytotoxic effects are dependent on the active proliferation of cells and rapid DNA replication to induce mismatches recognized by MMR machinery, which ultimately leads to either repair, death, or hypermutation (33). This implies that slower proliferation or dormancy would be protective against the acquisition of genomic hypermutation from TMZ treatment (Figure 1B).

There is accumulating evidence suggesting that solid tumors are driven by a minor population of stem cells (34). These multipotent stem cells give rise to rapidly proliferating progenitor cells, which are the major drivers of immediate tumor growth. Importantly, stem cells are phenotypically distinct from more differentiated progeny, characterized by their multipotent differentiation capacity, slower proliferation and greater drug efflux activity (34, 35). Together these likely contribute to the reportedly enhanced ability for stem cells to survive chemotherapy compared to their more differentiated progeny. Consistent with this, recent evidence has demonstrated that brain tumor stem cells (BTSCs) are responsible for initiating recurrence (36). Following completion of therapy, surviving stem populations exit dormancy and drives repopulation of the tumor mass. Notably, in this hierarchical model of tumor growth, only mutagenesis acquired by these stem cell populations will be represented in the recurrent tumor. Stem cells which emerge from dormancy following treatment result in recurrence with no observable hypermutation, an outcome independent from MGMT expression. It should also be noted that chemotherapy has been proposed to promote acquisition of stem-like features by differentiated cells (37, 38). As such, the traditional hierarchical model of differentiation and growth is likely insufficient to fully describe the disease state. Further investigation of this process of dedifferentiation is needed to understand its impact on the acquisition of hypermutation state at recurrence.

An additional feature which may impact the emergence of hypermutation is the concurrent use of radiation alongside TMZ for glioma. Indeed, radiation has been shown to be able to induce the expression of MGMT (39). Similarly, radiation has the capacity to drive transient growth arrest which as outlined above, may provide temporary resistance to TMZ induced mutagenesis (40). Whilst sufficiently powered datasets are not yet available for recurrent GBM to make definite conclusions regarding this, what is clear is that hypermutant tumors are able to emerge in patients which receive concurrent radiation and TMZ. We predict that generation of animal models exploring the modalities in clonal xenografts and assessment of incidence of hypermutation from using individual and combined treatment modalities will be able to elucidate the exact role of radiation in hypermutation and facilitate additional exploration into alternate means to drive evolution toward specific outcomes.

## Therapeutic Opportunities in Hypermutant Recurrent GBM

Both the diversity of genomic alterations as well as the underlying mechanisms which facilitate acquisition of hypermutation make it likely that the approach toward treating hypermutant tumors may be completely different from that of non-hypermutant recurrence. For example, the mutation of MMR genes is specifically observed in hypermutant but not non-hypermutant recurrent tumours (7, 22). Similarly, an increased neo-antigen load linked to the higher global tumor mutational burden is observed exclusively in hypermutant tumors. Exploitation of these unique features of recurrent GBM may provide the means to personalize patient treatment.

## Checkpoint Inhibitors

Immune checkpoint inhibitors (ICI) have shown great promise in the treatment of many diseases. High mutational burden has been identified as the best predictor of response to this treatment option, regardless of disease (41). This has culminated in the recent approval of the Programmed Death Protein 1 (PD-1) inhibitor pembrolizumab for use in all MMR-deficient or microsatellite instability (MSI)-high tumors. Of note, whilst the link between MMR-deficiency and hypermutation has been observed in several longitudinal studies (7, 20, 22), somewhat counter intuitively MSI is *not* associated with TMZ-mediated hypermutation in GBM (22). Regardless, case reports have suggested the capacity for ICI to be used for GBM in cases with hypermutation. For example, the treatment of GBM patients with germline POLE mutations driving a biallelic mismatch repair deficiency (bMMRd) phenotype characterized by hypermutation of the genome with pembrolizumab was reported to drive radiologically measured tumor regression (42, 43). However, the poor outcomes from the most recent Checkmate 143 trial (NCT02017717) which tested nivolumab and ipilimumab has moderated expectations with no improvement of survival in patients diagnosed with recurrent GBM. Importantly, this trial did not integrate any biomarkers of immunotherapy response such as mutational burden or T-cell inflamed gene expression profiling and as such, retrospective analyses following completion of this trial will likely be able to identify a subset of patients with the potential to respond to immunotherapy.

One feature of glioma which is thought to limit the impact of immunotherapy is the “cold” tumor immune environment of this disease. The blood brain barrier (BBB) comprises a system of pericytes, endothelial cells, astrocytic processes, and basement membrane, which has long been thought to prevent the movement of immune cells to the brain and contribute to the low immunogenicity of glioma. However, leukocyte trafficking across the BBB has been known to play an essential role in the control of several neurodegenerative and infectious diseases (44). Similarly, the paradigm of an intact BBB enforcing an immune privileged environment in GBM is increasingly being challenged as more evidence accumulates demonstrating that the BBB can be severely disrupted during disease in addition to observation of prevalent immune infiltration into the tumour (6, 45). As such, it is likely that the failure of immunotherapy is not limited by the BBB but instead due to intrinsic features of the local immune microenvironment such as immune-suppressive M2 macrophages or T-cell exhaustion which prevent robust immune surveillance and reactivity (46). Targeting these immunomodulatory cells is currently underway and may pave the way toward increasing the efficacy of immunotherapy in GBM.

## Combining Immunotherapy With Radiotherapy

A large number of studies have investigated the complex interaction between radiation and the immune system, which has led to the emergence of the radio-immunobiology (47). The most prominent example of this interaction is observation of the “abscopal effect” where irradiation against a primary tumor results in the regression of metastatic clones in disparate areas of the body. This is now understood to be mediated by the immune system: as tumor neo-antigens are released by dying cells, they are taken up by dendritic cells followed by systemic activation of T-cell responses which continue in a feedforward fashion leading to tumor control (48). It should be noted that induced immune response is greatly influenced by dose given per fraction, where increasing dose generates decreasing immune responses. For example, RT has been found to cause upregulation of immunosuppressive factors such as PD-L1 (49). Similarly, immunosuppressive M2 macrophages are more radioresistant than immune-promotive M1 macrophages and targeted radiation has been observed to cause a shift toward a M2 microenvironment in GBM (50). The complex role of radiation has now led to design of companion radio-immunotherapeutics (RIT) which acts to restrict the immunosuppressive effects of radiation. Furthermore, additional parameters such as the total dose of radiation, dose per fraction, and chronological sequencing of radiation and ICI warrant further investigation as to how they can be utilized to sustain immune cell infiltration into the tumor.

## Radiation and Hypermutation as a Synthetic Lethal Combination

In addition to the immunological relevance of hypermutation, the co-occurrence of MMR gene mutation in TMZ-driven hypermutant tumors may offer additional opportunities for exploitation (7, 20, 22). For example, PMS2 knockout cells demonstrate a 4-fold increase in mutations following treatment with radiation compared to their wild-type counterparts (51). Models of population fitness suggest that an increased mutation rate can be beneficial up to a point, beyond which further mutagenesis becomes detrimental due to accumulation of deleterious alterations. Accordingly, mouse models of genomic instability demonstrate a *decreased* tumor growth upon elevation of mutational burden (52). In the context of hypermutation, increasing the number of mutations in hypermutant cells following RT treatment may lead to reduced fitness, making them less aggressive and more amenable toward additional treatments.

## Conclusion and Future Perspectives on Hypermutation

It is now accepted that hypermutation represents a distinct subtype of recurrent glioma. However, several questions remain unresolved before we can start to understand the impact of hypermutation at recurrence. What is the mechanism behind emergence of hypermutation and is this process targetable? Is hypermutation in GBM associated with better or worse outcome for patients? Does immunotherapy represent a valid therapeutic approach for hypermutant tumors and can this be combined with radiotherapy? We predict that studies seeking to identify the underlying molecular features of hypermutant and non-hypermutant recurrent subtypes will likely pave the way for novel treatment approaches for recurrent GBM.

## Author Contributions

All authors listed have made a substantial, direct and intellectual contribution to the work, and approved it for publication.

### Conflict of Interest Statement

The authors declare that the research was conducted in the absence of any commercial or financial relationships that could be construed as a potential conflict of interest.
